# Efficacy of empagliflozin in heart failure with preserved versus mid-range ejection fraction: a pre-specified analysis of EMPEROR-Preserved

**DOI:** 10.1038/s41591-022-02041-5

**Published:** 2022-12-05

**Authors:** Stefan D. Anker, Javed Butler, Muhammad Shariq Usman, Gerasimos Filippatos, João Pedro Ferreira, Edimar Bocchi, Michael Böhm, Hans Pieter Brunner-La Rocca, Dong-Ju Choi, Vijay Chopra, Eduardo Chuquiure, Nadia Giannetti, Juan Esteban Gomez-Mesa, Stefan Janssens, James L. Januzzi, José R. González-Juanatey, Bela Merkely, Stephen J. Nicholls, Sergio V. Perrone, Ileana L. Piña, Piotr Ponikowski, Michele Senni, David Sim, Jindrich Spinar, Iain Squire, Stefano Taddei, Hiroyuki Tsutsui, Subodh Verma, Dragos Vinereanu, Jian Zhang, Tomoko Iwata, Janet M. Schnee, Martina Brueckmann, Stuart J. Pocock, Faiez Zannad

**Affiliations:** 1 Department of Cardiology (CVK) and Berlin Institute of Health Center for Regenerative Therapies (BCRT), German Centre for Cardiovascular Research (DZHK) partner site Berlin, Charité Universitätsmedizin Berlin, Berlin, Germany; 2 Institute of Heart Diseases, Wrocław Medical University, Wrocław, Poland; 3 Baylor Scott and White Research Institute, Dallas, TX USA; 4 University of Mississippi, Jackson, MS USA; 5 University of Mississippi Medical Center, Jackson, MS USA; 6 National and Kapodistrian University of Athens School of Medicine, Athens, Greece; 7 Université de Lorraine, INSERM, Centre d’Investigations Cliniques Plurithématique 1433, and INSERM U1116, CHRU, F-CRIN INI-CRCT (Cardiovascular and Renal Clinical Trialists), Nancy, France; 8 Cardiovascular Research and Development Center, Department of Surgery and Physiology, Faculty of Medicine of the University of Porto, Porto, Portugal; 9 Heart Failure Clinics, Instituto do Coracao, Hospital das Clinicas da Faculdade de Medicina da Universidade de São Paulo, Sao Paulo, Brazil; 10 Universitätsklinikum des Saarlandes, Homberg/Saar, Germany; 11 Maastricht University Medical Center, Maastricht, the Netherlands; 12 School for Cardiovascular Disease CARIM, Maastricht, the Netherlands; 13 Department of Medicine, Seoul National University Bundang Hospital, Seoul, South Korea; 14 Max Superspeciality Hospital, Saket, New Delhi, India; 15 National Institute of Cardiology, Mexico City, Mexico; 16 McGill University Health Centre, Montreal, Quebec Canada; 17 Cardiology Service, Fundación Valle del Lili, Universidad Icesi Cali, Cali, Colombia; 18 Department of Cardiovascular Diseases, University Hospitals Leuven, Leuven, Belgium; 19 Massachusetts General Hospital and Baim Institute for Clinical Research, Boston, MA USA; 20 Cardiology Department, University Hospital, CIBERCV, Santiago de Compostela, Spain; 21 Heart and Vascular Center, Semmelweis University, Budapest, Hungary; 22 Victorian Heart Institute, Monash University, Melbourne, Victoria Australia; 23 Argentine Catholic University, Buenos Aires, Argentina; 24 FLENI & IADT Institute, Buenos Aires, Argentina; 25 Central Michigan University, Mount Pleasant, MI USA; 26 Wrocław Medical University, Wrocław, Poland; 27 Cardiovascular Department, Cardiology Division, Papa Giovanni XXIII Hospital, Bergamo, Italy; 28 National Heart Centre Singapore, Singapore, Singapore; 29 Internal Cardiology Department, St Ann University Hospital and Masaryk University Brno, Brno, Czech Republic; 30 NIHR Biomedical Research Centre, University of Leicester, Glenfield Hospital, Leicester, UK; 31 Università di Pisa, Pisa, Italy; 32 Department of Cardiovascular Medicine, Faculty of Medical Sciences, Kyushu University, Fukuoka, Japan; 33 St. Michael’s Hospital, University of Toronto, Toronto, Ontario Canada; 34 University of Medicine and Pharmacy Carol Davila, Bucharest, Romania; 35 University and Emergency Hospital, Bucharest, Romania; 36 Heart Failure Center, Fuwai Hospital, Chinese Academy of Medical Science and Peking Union Medical College, Beijing, China; 37 Boehringer Ingelheim Pharma GmbH & Co. KG, Biberach, Germany; 38 Boehringer Ingelheim Pharmaceuticals Inc., Ridgefield, CT USA; 39 Boehringer Ingelheim International, Ingelheim, Germany; 40 First Department of Medicine, Faculty of Medicine Mannheim, University of Heidelberg, Mannheim, Germany; 41 London School of Hygiene and Tropical Medicine, London, UK; 42 Université de Lorraine, INSERM INI-CRCT, CHRU, Nancy, France

**Keywords:** Heart failure, Risk factors

## Abstract

The EMPEROR-Preserved trial showed that the sodium–glucose co-transporter 2 inhibitor empagliflozin significantly reduces the risk of cardiovascular death or hospitalization for heart failure (HHF) in heart failure patients with left ventricular ejection fraction (LVEF)  > 40%. Here, we report the results of a pre-specified analysis that separately evaluates these patients stratified by LVEF: preserved (≥ 50%) (*n* = 4,005; 66.9%) or mid-range (41–49%). In patients with LVEF  ≥ 50%, empagliflozin reduced the risk of cardiovascular death or HHF (the primary endpoint) by 17% versus placebo (hazard ratio (HR) 0.83; 95% confidence interval (CI): 0.71–0.98, *P* = 0.024). For the key secondary endpoint, the HR for total HHF was 0.83 (95%CI: 0.66–1.04, *P* = 0.11). For patients with an LVEF of 41–49%, the HR for empagliflozin versus placebo was 0.71 (95%CI: 0.57–0.88, *P* = 0.002) for the primary outcome (*P*_interaction_ = 0.27), and 0.57 (95%CI: 0.42–0.79, *P* < 0.001) for total HHF (*P*_interaction_ = 0.06). These results, together with those from the EMPEROR-Reduced trial in patients with LVEF < 40%, support the use of empagliflozin across the full spectrum of LVEF in heart failure.

## Abstract (web summary)

Analysis of the results of a large clinical trial, EMPEROR-Preserved, shows that the SGLT2 inhibitor empagliflozin improves outcomes for heart failure patients with either mid-range (41–49%) or preserved (≥50%) ejection fraction, supporting the use of this drug for both categories of patients.

## Main

Patients with heart failure have historically been classified into two groups based on their left ventricular ejection fraction (LVEF): heart failure with reduced ejection fraction (HFrEF) or heart failure with preserved ejection fraction (HFpEF). Although this dichotomous distinction has generally been useful in guiding contemporary management of heart failure, the exact LVEF cut-off demarcating HFpEF and HFrEF remains uncertain. Large-scale trials of drug interventions in patients with HFpEF have often used the LVEF inclusion criteria of >40% or >45% (ref. ^1^). Today, heart failure societies often classify LVEFs of 41–49% as ‘mildly reduced’ or ‘mid-range’ ejection fraction (HFmrEF)^2,3^. Recent guidelines consider patients with signs and symptoms of heart failure who present with objective evidence of cardiac structural and/or functional abnormalities along with an LVEF of ≥50% as having HFpEF^4^.

Although previous trials of patients with HFpEF failed to meet their primary endpoints, some trials appeared to show a positive signal. For instance, trials with angiotensin receptor–neprilysin inhibitor (ARNI), spironolactone and candesartan have reported modest but statistically non-significant reductions in the risk of the primary outcome of cardiovascular death or recurrent hospitalizations for heart failure in the overall HFpEF population^5–7^. Subgroup analyses showed that the treatment benefit was primarily seen in patients with HFmrEF, and that there was no significant benefit in the group of patients with HFpEF^5,8,9^.

EMPEROR-Preserved (Empagliflozin Outcome Trial in Patients with Chronic Heart Failure with Preserved Ejection Fraction) studied the effects of empagliflozin in patients with heart failure with an ejection fraction of >40% and identified a clinically meaningful and statistically significant effect on the primary endpoint of cardiovascular death or hospitalization for heart failure^10^. Given the effect modification by baseline LVEF seen in previous trials, the aim of this pre-specified analysis of the EMPEROR-Preserved trial was to document the effect of empagliflozin in patients with HFpEF (that is, LVEF ≥ 50%). We compare and contrast these results with the results derived from the patients who had HFmrEF (that is, an LVEF of 41–49%).

## Results

### Baseline characteristics

The EMPEROR-Preserved trial enrolled 5,988 patients. Two-thirds of the patients (*n* = 4,005; 66.9%) had HFpEF at baseline (that is, an LVEF ≥ 50%: 2,002 patients in the empagliflozin arm and 2,003 patients in the placebo arm). The remaining one-third (*n* = 1,983; 33.1%) had an LVEF of 41–49% (*n* = 995 in the empagliflozin arm and *n* = 988 in the placebo arm).

In the subgroup of patients with LVEF ≥ 50%, the average age of the participants was 73 ± 9 years, and half (50%) were women (Table 1). The mean age was 74 ± 9 years in women and 72 ± 9 years in men. The mean body mass index (BMI) in this group was 30 ± 6 kg m^−2^, and less than half of the participants (45%) had a history of smoking. The mean heart rate, systolic blood pressure and diastolic blood pressure were 70 ± 12 beats per minute, 133 ± 16 mmHg and 75 ± 11 mmHg, respectively. The majority of these participants had a history of hypertension (92%), and approximately half had a history of diabetes (48%), chronic kidney disease (56%) and atrial fibrillation or flutter (56%). The majority of the patients were treated with angiotensin-converting enzyme inhibitors (ACEIs) or angiotensin receptor blockers (ARBs) (77%), and beta-blockers (84%). One-third of the patients were treated with mineralocorticoid receptor antagonists (33%). For the patients with LVEF ≥ 50%, the baseline characteristics were balanced between the empagliflozin and placebo arms (Table 1).

**Table 1:** Baseline characteristics of participants with LVEF ≥ 50% or 41–49%, overall and by treatment group

	HFpEF (LVEF ≥ 50%)			HFmrEF (LVEF 41–49%)			*P*_HFpEF versus HFmrEF_
	Empagliflozin (*n* = 2,002)	Placebo (*n* = 2,003)	Overall (*n* = 4,005)	Empagliflozin (*n* = 995)	Placebo (*n* = 988)	Overall (*n* = 1,983)	
Age (years), mean (s.d.)	72.7 (9.2)	72.9 (9.2)	72.8 (9.2)	70.2 (9.3)	69.9 (10.0)	70.1 (9.7)	<0.001
Sex (self-reported), *n* (%)							
Female	1,016 (50.7)	1,003 (50.1)	2,019 (50.4)	322 (32.4)	335 (33.9)	657 (33.1)	<0.001
Male	986 (49.3)	1,000 (49.9)	1,986 (49.6)	673 (67.6)	653 (66.1)	1326 (66.9)	
BMI (kg m^−2^), mean (s.d.)	30.05 (5.97)	30.20 (6.00)	30.12 (5.98)	29.21 (5.44)	29.31 (5.73)	29.26 (5.58)	<0.001
Race, *n* (%)							
White	1,525 (76.2)	1,516 (75.7)	3,041 (75.9)	761 (76.5)	740 (74.9)	1501 (75.7)	0.003
Black or African American	84 (4.2)	75 (3.7)	159 (4.0)	49 (4.9)	50 (5.1)	99 (5.0)	
Asian	288 (14.4)	294 (14.7)	582 (14.5)	125 (12.6)	117 (11.8)	242 (12.2)	
Other (including mixed) or missing	105 (5.2)	118 (5.9)	223 (5.6)	60 (6.0)	81 (8.2)	141 (7.1)	
Region, *n* (%)							
North America	279 (13.9)	279 (13.9)	558 (13.9)	81 (8.1)	80 (8.1)	161 (8.1)	<0.001
Latin America	457 (22.8)	459 (22.9)	916 (22.9)	301 (30.3)	298 (30.2)	599 (30.2)	
Europe	890 (44.5)	890 (44.4)	1,780 (44.4)	456 (45.8)	453 (45.9)	909 (45.8)	
Asia	249 (12.4)	250 (12.5)	499 (12.5)	94 (9.4)	93 (9.4)	187 (9.4)	
Other	127 (6.3)	125 (6.2)	252 (6.3)	63 (6.3)	64 (6.5)	127 (6.4)	
Smoking status, *n* (%)							
Never smoked	1,108 (55.3)	1,073 (53.6)	2,181 (54.5)	470 (47.2)	494 (50.0)	964 (48.6)	<0.001
Ex-smoker	779 (38.9)	795 (39.7)	1,574 (39.3)	427 (42.9)	402 (40.7)	829 (41.8)	
Current smoker	114 (5.7)	134 (6.7)	248 (6.2)	97 (9.7)	91 (9.2)	188 (9.5)	
Missing	1 (<0.1)	1 (<0.1)	2 (<0.1)	1 (0.1)	1 (0.1)	2 (0.1)	
NYHA class, *n* (%)							
I	2 (0.1)	0	2 (<0.1)	1 (0.1)	1 (0.1)	2 (0.1)	0.58
II	1,624 (81.1)	1,631 (81.4)	3,255 (81.3)	808 (81.2)	820 (83.0)	1,628 (82.1)	
III	369 (18.4)	365 (18.2)	734 (18.3)	183 (18.4)	166 (16.8)	349 (17.6)	
IV	7 (0.3)	7 (0.3)	14 (0.3)	3 (0.3)	1 (0.1)	4 (0.2)	
Etiology of heart failure, *n* (%)							
Ischemic	587 (29.3)	547 (27.3)	1,134 (28.3)	492 (49.4)	491 (49.7)	983 (49.6)	<0.001
Hypertensive	831 (41.5)	859 (42.9)	1,690 (42.2)	235 (23.6)	261 (26.4)	496 (25.0)	
Other or missing	584 (29.2)	597 (29.8)	1,181 (29.5)	268 (26.9)	236 (23.9)	504 (25.4)	
NT-proBNP (pg ml^−1^), median (IQR)	981 (481–1,711)	909 (482–1,647)	946 (482–1,677)	1,013 (540–1,868)	1,037 (561–1,912)	1,025 (550–1,882)	<0.001
Heart rate (b.p.m.), mean (s.d.)	71 (12)	70 (12)	70 (12)	70 (12)	71 (12)	71 (12)	0.056
SBP (mmHg), mean (s.d.)	132 (16)	133 (16)	133 (16)	131 (16)	131 (15)	131 (15)	<0.001
DBP (mmHg), mean (s.d.)	76 (11)	75 (11)	75 (11)	76 (10)	77 (10)	76 (10)	<0.001
eGFR (ml min^−1^ 1.73 m^−2^), mean (s.d.)	59.4 (19.5)	59.5 (19.5)	59.4 (19.5)	63.1 (20.1)	63.0 (20.6)	63.0 (20.3)	<0.001
KCCQ-CSS, mean (s.d.)	69.7 (21.3)	69.2 (21.2)	69.5 (21.2)	71.2 (21.7)	73.5 (19.7)	72.4 (20.7)	<0.001
Medical history, *n* (%)							
Hypertension	1,837 (91.8)	1,831 (91.4)	3,668 (91.6)	884 (88.8)	872 (88.3)	1,756 (88.6)	<0.001
Diabetes	957 (47.8)	956 (47.7)	1,913 (47.8)	509 (51.2)	516 (52.2)	1,025 (51.7)	0.004
CKD (eGFR < 60 ml min^−1^ 1.73 m^−2^ or UACR > 300 mg g^−1^)	1,134 (56.6)	1,093 (54.6)	2,227 (55.6)	481 (48.2)	490 (49.6)	971 (49.0)	<0.001
Baseline hematocrit < median	950 (47.5)	964 (48.1)	2,089 (52.2)	396 (39.8)	414 (41.9)	810 (40.8)	<0.001
Atrial fibrillation or flutter	1,117 (55.8)	1,107 (55.3)	2,224 (55.5)	459 (46.1)	452 (45.7)	911 (45.9)	<0.001
Invasive electrophysiological procedure	123 (6.1)	141 (7.0)	264 (6.6)	38 (3.8)	46 (4.7)	84 (4.2)	<0.001
Valvular heart disease	341 (17.0)	313 (15.6)	654 (16.3)	141 (14.2)	124 (12.6)	265 (13.4)	0.003
Myocardial infarction	484 (24.2)	467 (23.3)	951 (23.7)	408 (41.0)	421 (42.6)	829 (41.8)	<0.001
PCI or CABG	575 (28.7)	554 (27.7)	1,129 (28.2)	399 (40.1)	386 (39.1)	785 (39.6)	<0.001
Heart failure medication, *n* (%)							
ACEIs/ARBs	1,561 (78.0)	1,537 (76.7)	3,098 (77.4)	806 (81.0)	801 (81.1)	1,607 (81)	0.001
ARNI	20 (1.0)	31 (1.5)	51 (1.3)	45 (4.5)	38 (3.8)	83 (4.2)	<0.001
Beta-blockers	1,688 (84.3)	1,687 (84.2)	3,375 (84.3)	910 (91.5)	882 (89.3)	1,792 (90.4)	<0.001
Ivabradine	22 (1.1)	17 (0.8)	39 (1.0)	18 (1.8)	14 (1.4)	32 (1.6)	0.031
MRAs	663 (33.1)	657 (32.8)	1,320 (33.0)	456 (45.8)	468 (47.4)	924 (46.6)	<0.001
Diuretics other than MRA	1,620 (80.9)	1,626 (81.2)	3,246 (81.0)	787 (79.1)	776 (78.5)	1,563 (78.8)	0.041
Triple therapy^a^	443 (22.1)	440 (22.0)	883 (22.0)	372 (37.4)	371 (37.6)	743 (37.5)	<0.001
ICD or CRT-D, *n* (%)	58 (2.9)	70 (3.5)	128 (3.2)	55 (5.5)	49 (5.0)	104 (5.2)	0.002

^a^Defined as: (ACEI or ARB or ARNI) + (beta-blocker or ivabradine) + MRA.

*P* values are two-sided and are derived from *t*-tests for continuous variables and chi-squared tests for categorical variables. No adjustments for multiple testing were made. ACEI, angiotensin-converting enzyme inhibitor; ARB, angiotensin receptor blocker; ARNI, angiotensin receptor–neprilysin inhibitor; BMI, body mass index; CABG, coronary artery bypass graft; CKD, chronic kidney disease; CRT-D, cardiac resynchronization therapy defibrillator; DBP, diastolic blood pressure; eGFR, estimated glomerular filtration rate; ICD, implantable cardioverter defibrillator; KCCQ-CSS, Kansas City Cardiomyopathy Questionnaire–Clinical Summary Score; LVEF, left ventricular ejection fraction; MRA, mineralocorticoid receptor antagonist; NT-proBNP, N-terminal pro-brain natriuretic peptide; NYHA, New York Heart Association; PCI, percutaneous coronary intervention; SBP, systolic blood pressure; UACR, urinary albumin-to-creatine ratio.

The baseline characteristics of the patients with LVEF ≥ 50% differed considerably from those of the patients with LVEF 41–49% (Table 1). Patients with LVEF ≥ 50% were significantly more likely to be older, be women, and have a higher BMI. These patients had a higher burden of hypertension, chronic kidney disease, atrial fibrillation and valvular heart disease. In contrast, they were significantly less likely to have a history of diabetes or myocardial infarction. Baseline New York Heart Association (NYHA) functional class did not differ by LVEF category, however, patients with LVEF ≥ 50% were more likely to have lower N-terminal pro-brain natriuretic peptide levels. These patients had lower mean baseline Kansas City Cardiomyopathy Questionnaire–Clinical Summary (KCCQ-CS) scores. Baseline use of heart failure medications (including ACEIs, ARBs, ARNI, beta-blockers and mineralocorticoid receptor antagonists) was lower in patients with LVEF ≥ 50%.

### Efficacy of empagliflozin according to baseline LVEF

**Fig. 1:**
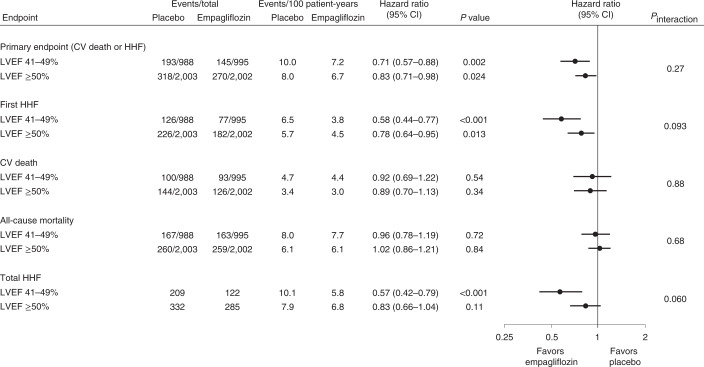
Effect of empagliflozin versus placebo on time-to-first-event outcomes and total heart failure hospitalizations by LVEF category. Hazard ratios for the primary endpoint, first hospitalization for heart failure (HHF), cardiovascular (CV) death and all-cause mortality were calculated using a multivariable Cox regression model, whereas the hazard ratio for total HHF was calculated using a joint frailty model with CV death as competing risk, as described in the Methods. Data are presented as point estimates and 95% CIs with two-sided *P* values. No adjustments for multiple testing were made.

**Fig. 2:**
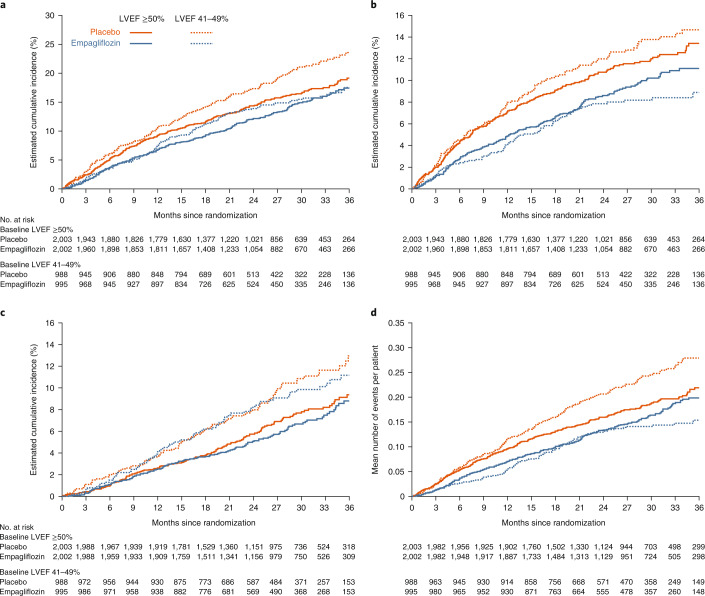
Comparison of effects of empagliflozin versus placebo on outcomes by LVEF category. Effects are shown for the primary outcome (cardiovascular death or hospitalization for heart failure) (**a**), first hospitalization for heart failure (**b**), cardiovascular death (**c**) and total hospitalizations for heart failure (**d**). Detailed results for all related modeled analyses are shown in Fig. 1.

In the subgroup of patients with LVEF ≥ 50%, the primary outcome of the composite of cardiovascular death or hospitalization for heart failure occurred in 270 participants (13.5%) in the empagliflozin group and in 318 participants (15.9%) in the placebo group. Empagliflozin treatment resulted in a statistically significant reduction in the risk of the primary outcome by 17% compared with placebo (270 of 2,002, 6.7 per 100 patient-years versus 318 of 2,003, 8.0 per 100 patient-years, respectively; hazard ratio (HR) 0.83, 95% confidence interval (CI): 0.71–0.98, *P* = 0.024; Figs. 1 and 2a). When the components of the primary outcome were analyzed separately for patients with LVEF ≥ 50%, empagliflozin was found to significantly reduce the first hospitalizations for heart failure by 22% compared with placebo (182 of 2,002, 4.5 per 100 patient-years versus 226 of 2,003, 5.7 per 100 patient-years; HR 0.78, 95% CI: 0.64–0.95, *P* = 0.013), but not cardiovascular mortality (126 of 2,002, 3.0 per 100 patient-years versus 144 of 2,003, 3.4 per 100 patient-years; HR 0.89, 95% CI: 0.70–1.13, *P* = 0.34; Figs. 1 and 2b,c).

For patients with LVEF 41–49% the effect size of empagliflozin compared with placebo for the primary outcome of EMPEROR-Preserved was 29% (145 of 995, 7.2 per 100 patient-years versus 193 of 988, 10.0 per 100 patient-years; HR 0.71, 95% CI: 0.57–0.88, *P* = 0.002; Figs. 1 and 2), and it was 42% for first hospitalizations for heart failure (77 of 995, 3.8 per 100 patient-years versus 126 of 988, 6.5 per 100 patient-years; HR 0.58, 95% CI: 0.44–0.77, *P* < 0.001; Fig. 1), without an effect on cardiovascular mortality (93 of 995, 4.4 per 100 patient-years versus 100 of 988, 4.7 per 100 patient-years; HR 0.92, 95% CI: 0.69–1.22, *P* = 0.54; Fig. 1).

The effect of empagliflozin versus placebo did not significantly differ between patients with LVEF 41–49% and ≥ 50% for the primary outcome, first hospitalization for heart failure, or for cardiovascular mortality (*P* values for the interaction between treatment and baseline LVEF category of 0.27, 0.09 and 0.88, respectively).

The HR for the effect of empagliflozin on first and recurrent hospitalization for heart failure was 0.83 (95% CI: 0.66–1.04, *P* = 0.11) in patients with LVEF ≥ 50% and 0.57 (95% CI: 0.42–0.79, *P* < 0.001) in patients with LVEF 41–49% (*P* = 0.06 for the interaction between treatment and baseline LVEF category, Figs. 1 and 2d).

The number needed to treat to prevent a first hospitalization for heart failure with empagliflozin compared with placebo over 2.15 years of treatment was 44 (95% CI: 24–248) and 20 (95% CI: 13–40) in the LVEF ≥ 50% and LVEF 41–49% groups, respectively. For total hospitalizations for heart failure, the number needed to treat was 38 (95% CI: 15–68) in the LVEF ≥ 50% group and 9 (95% CI: 6–25) in the LVEF 41–49% group.

**Fig. 3:**
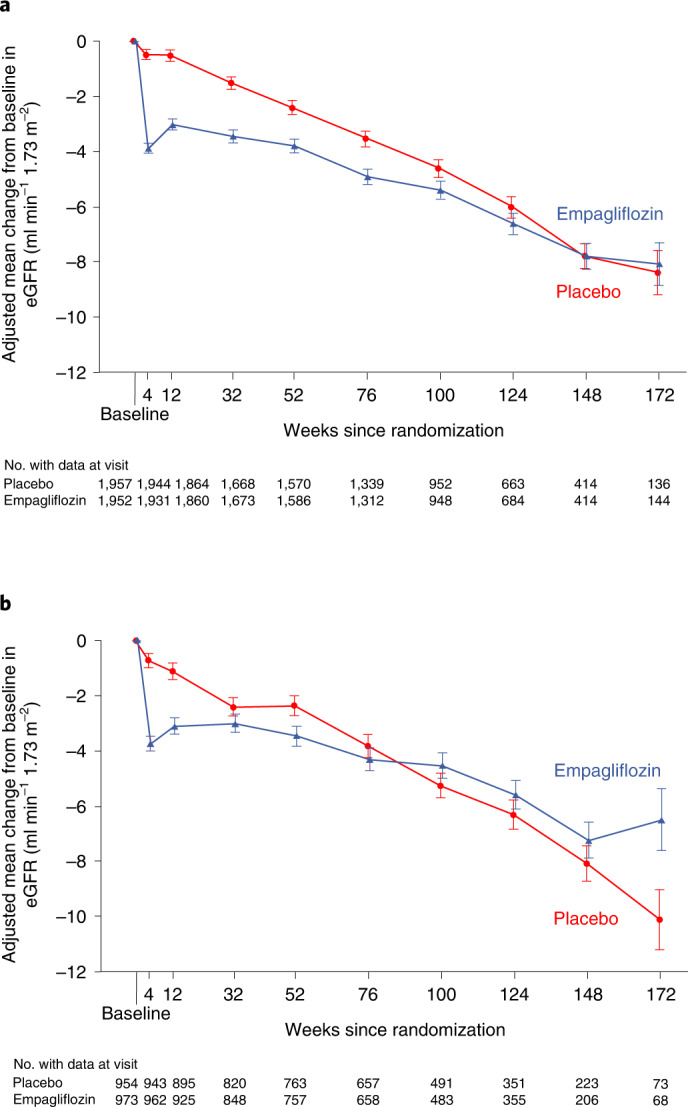
Comparison of empagliflozin versus placebo for change in eGFR over time and eGFR slope by LVEF category. Effects are shown for LVEF ≥ 50% (**a**) and LVEF 41–49% (**b**). **a**, Between-group difference in slope: 1.24 ml min^−1^ 1.73 m^−2^ per year (95% CI: 0.87–1.61, *P* < 0.0001). **b**, Between-group difference in slope: 1.61 ml min^−1^ 1.73 m^−2^ per year (95% CI: 1.09–2.13, *P* < 0.0001). Data are presented as adjusted mean and standard error. Change in eGFR was analyzed using a mixed model for repeated measures while the eGFR slope (that is, the rate of change in the decrease in eGFR) was analyzed using a random coefficient model, as described in the Methods.

Empagliflozin slowed the decline in slope of estimated glomerular filtration rate (eGFR) in the patients with LVEF ≥ 50% by 1.24 ml min^−1^ 1.73 m^−2^ per year (95% CI: 0.87–1.61, *P* < 0.001; Fig. 3a) and in the patients with LVEF 41–49% by 1.61 ml min^−1^ 1.73 m^−2^ per year (95% CI: 1.09–2.13, *P* < 0.001; Fig. 3b). The treatment effect was similar in the two subgroups (*P* = 0.25 for heterogeneity across subgroups).

Empagliflozin had no significant effect on time to all-cause mortality in patients with LVEF ≥ 50% (HR 1.02; 95% CI: 0.86–1.21, *P* = 0.84) or in patients with LVEF 41–49% (HR 0.96; 95% CI: 0.78–1.19, *P* = 0.72; *P* = 0.68 for the interaction between treatment and baseline LVEF category).

**Fig. 4:**
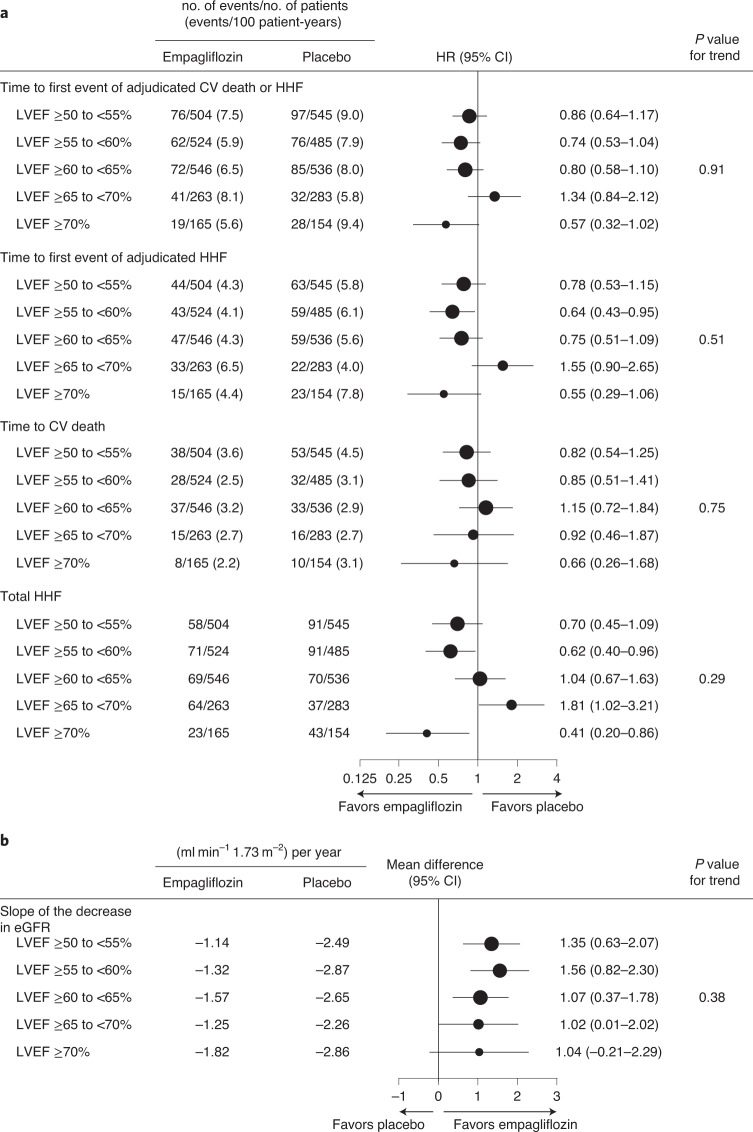
Comparison of empagliflozin versus placebo for outcomes by LVEF subgroups in patients with LVEF ≥ 50%. Effects are shown for the first event of cardiovascular (CV) death or hospitalization for heart failure (HHF), first HHF, CV death and total HHF (**a**), and the slope of change in eGFR (**b**) for patients in subgroups of LVEF from 50% to 70%. Data for the clinical events are presented as point estimates and 95% confidence intervals (CIs); data for the difference in slope of eGFR are presented as mean values and 95% CIs.

Figure 4 shows the results for the primary endpoint and its components as well as the total (first and recurrent) hospitalizations for heart failure and eGFR slope by 5% increments of LVEF for patients in EMPEROR-Preserved with an LVEF ≥ 50%.

**Extended Data Fig. 1:**
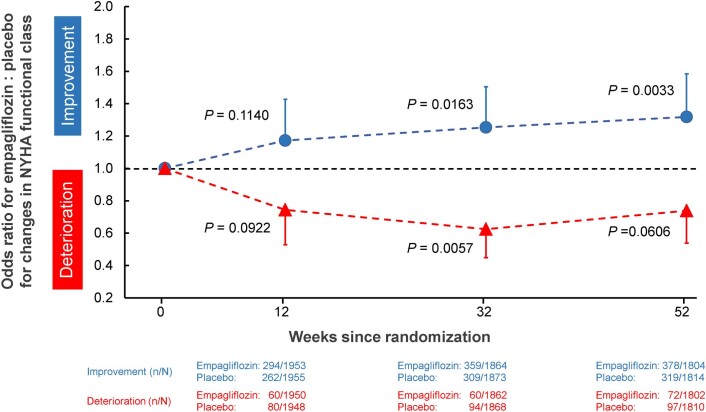
Effect of empagliflozin versus placebo on changes in NYHA functional class in patients with LVEF ≥ 50%. Odds ratios were calculated using a multivariable Cox regression model. Data are presented as point estimates and 95% CIs with two-sided *P* values. No adjustments for multiple testing were made. Patients treated with empagliflozin had higher odds of improving NYHA class at week 52 (odds ratio 1.32 [95% CI: 1.10–1.56]; *P* = 0.0033) and lower odds of worsening NYHA class at week 52 (odds ratio 0.74 [95% CI: 0.54–1.01]; *P* = 0.0606). LVEF, left ventricular ejection fraction; NYHA, New York Heart Association.

In the overall trial cohort of EMPEROR-Preserved, empagliflozin significantly improved mean KCCQ-CS score from baseline compared with placebo at weeks 12, 32 and 52 (adjusted mean differences of 1.03 (95% CI: 0.32–1.74), 1.24 (95% CI: 0.44–2.04) and 1.50 (95% CI: 0.64–2.36), respectively)^11^. This effect was consistent between participants with LVEF ≥ 50% and those with LVEF 41–49% (all *P*_interaction_ ≥ 0.35) (Table 2). Similar findings were seen when KCCQ total summary score and overall summary score were studied (Table 2). Patients with LVEF ≥ 50% had a 34% higher likelihood of being in a lower NYHA class at week 52 (*P* < 0.001) when treated with empagliflozin. Patients treated with empagliflozin had higher odds of improving NYHA class at week 52 (odds ratio (OR) 1.32; 95% CI: 1.10–1.56, *P* = 0.0033) and lower odds of worsening NYHA class at week 52 (OR 0.74; 95% CI: 0.54–1.01, *P* = 0.0606) (Extended Data Fig. 1).

**Table 2:** Treatment effect on KCCQ summary scores by LVEF category

	LVEF 41–49% (*n* = 1,983)			*P* value	LVEF ≥ 50% (*n* = 4,005)			*P* value	*P*_interaction_^a^
	Empagliflozin (*n*)	Placebo (*n*)	Difference between empagliflozin and placebo		Empagliflozin (n)	Placebo (n)	Difference between empagliflozin and placebo		
KCCQ-CSS, mean change from baseline (95% CI)									
Week 12	943	909	0.54 (−0.70–1.78)	0.39	1,903	1,908	1.27 (0.40–2.14)	0.004	0.35
Week 32	867	842	1.21 (−0.19–2.62)	0.090	1,749	1,734	1.24 (0.26–2.22)	0.013	0.97
Week 52	801	795	1.56 (0.05–3.06)	0.043	1,672	1,662	1.46 (0.42–2.51)	0.006	0.92
KCCQ-TSS, mean change from baseline (95% CI)									
Week 12	943	909	1.24 (−0.14–2.62)	0.078	1,903	1,908	2.01 (1.05–2.98)	<0.001	0.37
Week 32	867	842	1.33 (−0.20–2.85)	0.088	1,749	1,733	1.60 (0.54–2.67)	0.003	0.77
Week 52	801	795	1.91 (0.29–3.53)	0.021	1,671	1,662	2.14 (1.02–3.26)	<0.001	0.82
KCCQ-OSS, mean change from baseline (95% CI)									
Week 12	943	909	0.13 (−1.10–1.36)	0.84	1,903	1,908	1.57 (0.71–2.43)	<0.001	0.060
Week 32	867	842	1.13 (−0.24–2.51)	0.11	1,749	1,734	1.72 (0.76–2.68)	<0.001	0.49
Week 52	801	795	1.55 (0.08–3.03)	0.039	1,672	1,662	1.63 (0.60–2.65)	0.002	0.93

*P* values are two-sided. No adjustments for multiple testing were made. Data are based on a mixed model with repeated measures that included age and baseline eGFR as linear covariates, and region, diabetes status, sex, week reachable, visit-by-treatment-by-LVEF subgroup interaction and baseline KCCQ summary score-by-visit interaction as fixed effects.

eGFR, estimated glomerular filtration rate; LVEF, left ventricular ejection fraction; KCCQ, Kansas City Cardiomyopathy Questionnaire; CSS, Clinical Summary Score; TSS, Total Summary Score; OSS, Overall Summary Score.

^a^Interaction of treatment by LVEF category

## Discussion

In patients with heart failure and LVEF ≥ 50%, empagliflozin significantly reduced the risk of cardiovascular death or hospitalization for heart failure by 17%. This was predominantly driven by a reduction in the risk of first hospitalization for heart failure. Empagliflozin significantly reduced the rate of decline in eGFR and also improved health-related quality of life and functional class in these patients. The participants with LVEF ≥ 50% had some notably different clinical characteristics compared with those with LVEF < 50%, in that they were older, had a different burden of comorbidities (including lower incidence of previous myocardial infarction and ischemic etiology of heart failure, and higher incidence of kidney disease) and were more likely to be women. Participants with LVEF ≥ 50% also had lower quality of life (lower mean KCCQ score).

**Extended Data Fig. 2:**
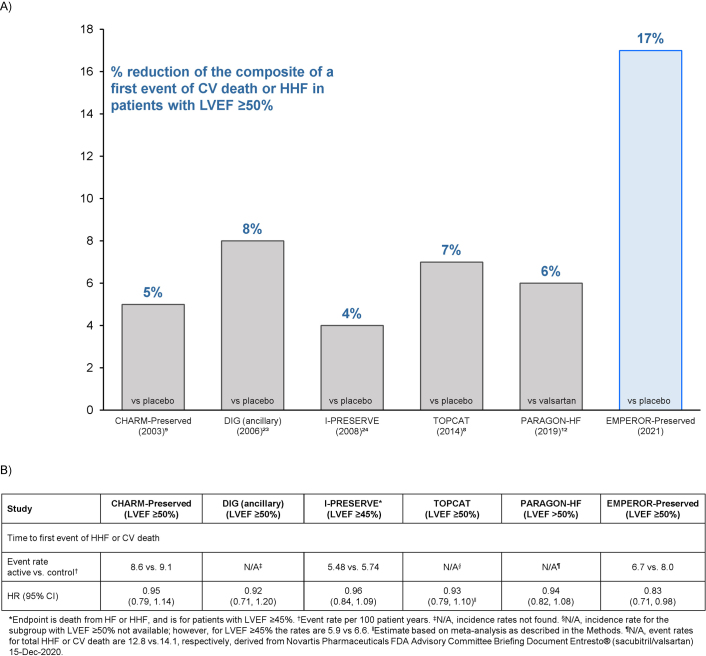
The effect of different heart failure therapies tested in specific trials aiming to recruit HFpEF patients. Known and estimated treatment effects for the composite endpoint of the time to a first event of cardiovascular death or HHF are displayed for the subgroup of patients with LVEF ≥ 50%. Panel A shows treatment effect sizes, and Panel B provide hazard ratios and event rates as are available. CI, confidence interval; CV, cardiovascular; HF, heart failure; HHF, hospitalization for heart failure; HR, hazard ratio; LVEF, left ventricular ejection fraction.

Several other trials have assessed therapies in patients with HFpEF (Extended Data Fig. 2). The CHARM (Candesartan in Heart Failure: Assessment of Reduction in Mortality and Morbidity) program included 1,953 patients with true HFpEF (LVEF ≥ 50%)^9^. In these patients, candesartan did not reduce the composite of cardiovascular death or hospitalization for heart failure (HR 0.95; 95% CI: 0.79–1.14, *P* = 0.57). The TOPCAT (Treatment of Preserved Cardiac Function Heart Failure With an Aldosterone Antagonist) trial of spironolactone included 2,924 patients with LVEF ≥ 50%^8^. No benefit for cardiovascular death or hospitalization for heart failure for spironolactone versus placebo was shown in these individuals (estimated HR 0.93; 95% CI: 0.79–1.10). Similarly, in the PARAGON-HF (Prospective Comparison of ARNI with ARB Global Outcomes in HF with Preserved Ejection Fraction) trial^5^, the combination of sacubitril and valsartan did not reduce cardiovascular death or hospitalization for heart failure in the subgroup of 4,067 patients with LVEF > 50% (HR 0.94; 95% CI: 0.82–1.08, *P* = 0.38)^12^. In direct comparison with the latter trial, the HR for cardiovascular death or hospitalization for heart failure with empagliflozin versus placebo was 0.82 (95% CI: 0.69–0.98, *P* = 0.026) in the 3,501 patients in EMPEROR-Preserved with LVEF > 50% (a subset of those with LVEF ≥ 50%) (Extended Data Table 1).

Of note, the effect of empagliflozin versus placebo on total (first and recurrent) hospitalizations for heart failure has been shown to be consistent in most of the pre-specified subgroups, but an interaction between treatment and LVEF (*P*_trend_ = 0.008) was observed for this endpoint, with an attenuated response in patients with an LVEF ≥ 60% considering data from EMPEROR-Preserved^13,14^ or from EMPEROR-Pooled^15^. Here, it is shown that the effect of empagliflozin versus placebo is less pronounced in patients with LVEF ≥ 50% than in patients with LVEF 41–49% for first hospitalization for heart failure (22% versus 42% reduction, respectively; *P*_interaction_ = 0.09), and for total hospitalizations for heart failure (17% versus 43%; *P*_interaction_ = 0.06), which would support this previous observation. For the primary endpoint the risk reduction with empagliflozin in the two LVEF subgroups was 17% in patients with LVEF ≥ 50% and 29% in patients with an LVEF of 41–49% (*P*_interaction_ = 0.27).

For sotagliflozin a significant reduction in the composite endpoint of cardiovascular death, total hospitalizations for heart failure, or urgent visits for heart failure was demonstrated in heart failure patients with an LVEF ≥ 50% who all had diabetes in a pooled analysis of 739 patients from the SCORED (Sotagliflozin on Cardiovascular and Renal Events in Patients with Type 2 Diabetes and Moderate Renal Impairment Who Are at Cardiovascular Risk) and SOLOIST-WHF (Sotagliflozin on Cardiovascular Events in Patients with Type 2 Diabetes Post Worsening Heart Failure) trials^16^. Neither SCORED nor SOLOIST-WHF was a specific HFpEF study; the trials were stopped early; the analyses were post hoc; and the population studied represented <8% of the total number of enrolled patients. The PRESERVED HF study showed that dapagliflozin improved patient-reported symptoms, physical limitations and exercise function in patients with LVEF ≥ 45%^17^. The DELIVER (Dapagliflozin Evaluation to Improve the Lives of Patients with Preserved Ejection Fraction Heart Failure) study^18^ will be important to establish whether improvements in outcomes in heart failure patients with LVEF ≥ 50% could be a class effect of sodium–glucose co-transporter 2 (SGLT2) inhibitors.

Early trials studying treatments for HFrEF included patients with LVEFs of <35% or <40%. By contrast, early HFpEF trials often used an LVEF cut-off of >45% or >50%. This resulted in patients with HFmrEF (LVEF 41–49%) being poorly studied in trials^19^. The present analysis shows that empagliflozin significantly reduces the composite outcome of cardiovascular death or hospitalization for heart failure as well as first hospitalization for heart failure in patients with an LVEF between 41% and 49%. Thus, although patients with mildly reduced LVEF may have clinical characteristics that are intermediate between classic HFrEF and HFpEF, these patients seem to respond to the four foundational treatments of heart failure therapy in a fashion similar to patients with HFrEF. Thus, designation of HFmrEF as a separate category to HFrEF may not be relevant from a clinical perspective^20^.

Finally, empagliflozin slowed the decline in eGFR to a similar extent in patients with LVEF ≥ 50% and those with LVEF 41–49%. In both subgroups an initial decrease compared with placebo was followed by slower long-term decline, an effect that has been consistently observed with SGLT2 inhibitors.

This study has certain strengths and limitations. Notably, it was a pre-specified analysis of the largest randomized, double-blind trial of a drug intervention for heart failure in patients with LVEF > 40%. However, ejection fraction was not measured in a central laboratory and thus was subject to the normal variability of clinical practice. Furthermore, interpretation of treatment effect in the LVEF ≥ 50% group compared with the LVEF 41–49% group was based on a cut-off of 0.05 for interaction *P* values. Finally, our comparison of the efficacy of different therapies in patients with LVEF ≥ 50% (or similar ejection fraction range) should be interpreted cautiously, given the differences in patient characteristics and study design across trials.

In conclusion, in this subgroup analysis of the EMPEROR-Preserved trial, empagliflozin significantly improved the composite of cardiovascular death or hospitalization for heart failure in patients with HFpEF with LVEF ≥ 50% (relative reduction versus placebo of 17%); however, the treatment effect appeared to be less pronounced compared with patients with LVEF 41–49% (relative reduction, 29%), although the difference was not statistically significant (*P* = 0.27). This benefit was driven largely by a reduction in hospitalizations for heart failure (number needed to treat over 2.2 years: 44 and 20 in patients with LVEF ≥ 50% and LVEF 41–49%, respectively), but empagliflozin also improved health-related quality of life and functional class. These observations represent the first demonstration of a clinically meaningful and statistically significant improvement for any drug in patients with HFpEF who have an LVEF ≥ 50%, and when considered together with the results of EMPEROR-Reduced, our findings support the use of empagliflozin across the full spectrum of ejection fractions in patients with heart failure.

## Methods

### Study design

The design and primary results of the EMPEROR-Preserved trial (ClinicalTrials.gov identifier: NCT03057951) have been published previously^10,21^. Ethics approval was obtained at each study site, and all patients provided informed consent to participate in the study. Data will be made available on request in adherence with transparency conventions in medical research and through requests to the corresponding author. The executive committee of EMPEROR has developed a comprehensive analysis plan and numerous pre-specified analyses, which will be presented in future scientific meetings and publications. At a later point in time, the full database will be made available in adherence with the transparency policy of the sponsor (available at https://trials.boehringer-ingelheim.com/transparency_policy.html).

EMPEROR-Preserved was a double-blind, randomized, placebo-controlled, and event-driven clinical trial designed to assess the safety and efficacy of empagliflozin for the treatment of HFpEF. Key inclusion criteria included chronic heart failure (NYHA class II–IV), an LVEF of >40% (and no prior measurement of LVEF ≤ 40% under stable conditions), an elevated N-terminal prohormone B-type natriuretic peptide level at screening of >300 pg ml^−1^ (>900 pg ml^−1^ for patients with baseline atrial fibrillation), and either hospitalization for heart failure in the past 12 months or structural abnormalities on echocardiography (left atrial enlargement or left ventricular hypertrophy). A total of 5,988 participants were enrolled and randomly assigned (in a 1:1 manner) to receive either empagliflozin 10 mg or placebo, in addition to usual therapy. Randomization was stratified by the following variables: LVEF ≥ or <50; diabetes status at screening; eGFR ≥ or <60 ml min^−1^ 1.73 m^−2^; and geographical region (North America, Latin America, Europe, Asia, and other). Participants were followed for the occurrence of pre-specified clinical outcomes for the entire duration of the trial, regardless of adherence to study protocol, unless consent was withdrawn or the participant was lost to follow-up. The median follow-up time in EMPEROR-Preserved was 26.2 months (interquartile range, 18.1–33.1).

### Categorization of ejection fraction at baseline

Baseline LVEF was to be determined during screening using the most recent assessment in the past 6 months or an assessment during screening. LVEF assessment using echocardiography, radionuclide ventriculography, invasive angiography, magnetic resonance imaging or computed tomography was acceptable. For the present analysis, patients were categorized into two groups based on baseline LVEF in which true HFpEF was defined as LVEF ≥ 50% and HFmrEF was defined as LVEF 41–49%, according to the 2021 European Society of Cardiology guidelines on heart failure^4^. A known prior LVEF of ≤40% was an exclusion criterion for recruitment into the EMPEROR-Preserved study. Of note, two patients with baseline LVEF of 40% were included in the trial and included in the group of LVEF 41–49%.

### Outcomes of interest

The following clinical outcomes were of interest in the present study: (1) the primary composite endpoint in EMPEROR-Preserved of the time to cardiovascular death or a first event of hospitalization for heart failure; (2) first hospitalization for heart failure; (3) cardiovascular mortality (both (2) and (3) were secondary endpoints of EMPEROR-Preserved); (4) total (first and recurrent) hospitalization for heart failure; and (5) the rate of change in the eGFR slope (both (4) and (5) were key secondary endpoints). The change in health-related quality of life was assessed using the KCCQ-23^22^, which was completed at randomization and at 12, 32 and 52 weeks of follow-up. All three summary scores of the KCCQ-23 were evaluated: the total symptom score (TSS), which quantifies symptom severity and frequency; the clinical summary score (CSS), which consists of the symptom and physical function domains; and the overall summary score (OSS), which includes the CSS as well as the quality of life and social limitation domains. In addition, NYHA class was analyzed at baseline and at week 52.

### Statistical analysis

All clinical data were captured using the electronic data capture system RAVE. SAS v9.4 was used for all analyses. Baseline characteristics of patients in each LVEF category (41–49% and ≥ 50%) were analyzed descriptively. Categorical variables were summarized as frequencies and percentages and compared between the two LVEF categories using the chi-squared test, while continuous variables were summarized as means and standard deviations and compared using the *t*-test.

All outcomes were analyzed according to the intention-to-treat principle. The effect of empagliflozin versus placebo on time-to-first-event outcomes was analyzed using a multivariable Cox regression model and presented as HRs and 95% CIs. The effect of empagliflozin on total hospitalizations for heart failure was analyzed using a joint frailty model, with cardiovascular death as competing risk. In both cases the multivariable models were adjusted for the following baseline characteristics: age, sex, eGFR, diabetes status, and region. The number needed to treat to prevent one event per 2.15 years at risk was calculated using the exponential distribution for the first event and the negative binomial model for recurrent events.

As pre-specified, the change in eGFR slope was analyzed based on on-treatment data, using a random coefficient model that enabled the intercept and gradient to vary randomly between patients. The analysis model included age, baseline eGFR, sex, diabetes status, region, baseline eGFR × time interaction, treatment × LVEF subgroup interaction and time × treatment × LVEF subgroup interaction as covariates. Change in eGFR over time was analyzed using a mixed model for repeated measures that included age, sex, diabetes status, region, week reachable, time × treatment × LVEF subgroup interaction and baseline eGFR × time interaction as covariates.

KCCQ summary scores (TSS, CSS and OSS) were analyzed using a mixed model with repeated measures. This model included age and baseline eGFR as linear covariates and region, diabetes status, sex, week reachable, visit × treatment × LVEF subgroup interaction and baseline KCCQ summary score × visit interaction as fixed effects. NYHA functional class was analyzed using a partial proportional odds regression model adjusted for the same variables used in the Cox regression model and baseline NYHA class, assuming proportionality for all covariates except region and baseline NYHA class. In addition, improvement and deterioration of NYHA class were analyzed using logistic regression with the same covariates.

Consistency of treatment effects across the two LVEF groups was evaluated by adding subgroup × treatment interaction terms to the models. Results with two-sided *P* < 0.05 are described as statistically significant. No adjustments for multiple testing were made.

For comparisons with other trials, time to cardiovascular death or first hospitalization for heart failure (or similar endpoint) in HFpEF patients was taken from published data for the CHARM-Preserved^9^, DIG^23^, I-Preserved^24^ and PARAGON-HF^12^ trials. For the TOPCAT trial, published data for LVEF 50–54.99% (HR 0.85, 95% CI: 0.61–1.18), LVEF 55–59.99% (HR 0.94, 95% CI: 0.68–1.29) and LVEF ≥ 60% (HR 0.97, 95% CI: 0.76–1.23)^8^ were meta-analyzed using a fixed-effects model to derive a pooled HR of 0.93 (95% CI: 0.79–1.10) for LVEF ≥ 50%.

### Reporting summary

Further information on research design is available in the Nature Research Reporting Summary linked to this article.

## Online content

Any methods, additional references, Nature Research reporting summaries, source data, extended data, supplementary information, acknowledgements, peer review information; details of author contributions and competing interests; and statements of data and code availability are available at 10.1038/s41591-022-02041-5.

### Supplementary information

Reporting Summary

## Figures and Tables

**Extended Data Table 1:**
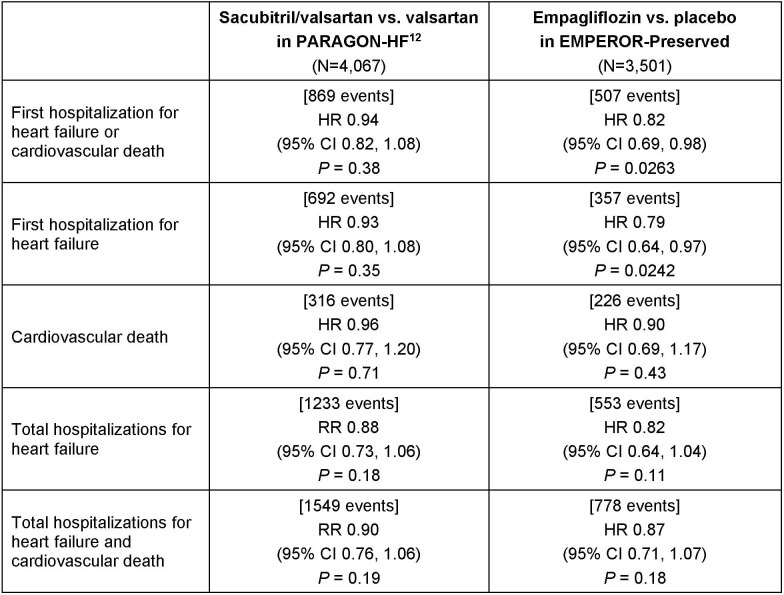
Effect of empagliflozin versus placebo on clinical outcomes in the EMPEROR-Preserved trial and of sacubitril/valsartan versus valsartan in the PARAGON-HF trial^12^ in patients with left ventricular ejection fraction >50%

Data are presented as point estimates and 95% Cls. Data for sacubitril/valsartan were calculated as described by Solomon et al^12^ (including the method of Lin et al^25^ to analyze total HHF and total HHF and CV death). For empagliflozin, HRs for first HHF or CV death, first HHF, and CV death were calculated using a multivariable Cox regression model while the HR for total HHF was calculated using a joint frailty model (both described in the Methods) and the HR for total HHF and CV death was calculated according to Lin et al^25^; *P* values are two-sided and no adjustments for multiple testing were made. CI, confidence interval; CV, cardiovascular; HHF, hospitalization for heart failure, HR, hazard ratio; RR, rate ratio.

## Data Availability

To ensure independent interpretation of clinical study results and enable authors to fulfill their role and obligations under the ICMJE criteria, Boehringer Ingelheim grants all external authors access to clinical study data pertinent to the development of the publication. In adherence with the Boehringer Ingelheim Policy on Transparency and Publication of Clinical Study Data, scientific and medical researchers can request access to clinical study data when it becomes available on https://vivli.org/, and earliest after publication of the primary manuscript in a peer-reviewed journal, regulatory activities are complete, and other criteria are met. Please visit https://www.mystudywindow.com/msw/datasharing for further information.

## Extended data

are available for this paper at 10.1038/s41591-022-02041-5.
