# Supplementary material for: Efficacy of empagliflozin in heart failure with preserved versus mid-range ejection fraction: a pre-specified analysis of EMPEROR-Preserved
Source: Nat Med. 2022 Dec 5;28(12):2512–20. doi: 10.1038/s41591-022-02041-5 (PMC9800272; doi:10.1038/s41591-022-02041-5)
Supplement: Supplementary file 1 — Reporting Summary [file 41591_2022_2041_MOESM1_ESM.pdf]

nature portfolio

Corresponding author(s): Stefan D. Anker

Last updated by author(s): Aug 16, 2022

Reporting Summary

Nature Portfolio wishes to improve the reproducibility of the work that we publish. This form provides structure for consistency and transparency in reporting. For further information on Nature Portfolio policies, see our Editorial Policies and the Editorial Policy Checklist.

Statistics

For all statistical analyses, confirm that the following items are present in the figure legend, table legend, main text, or Methods section.

n/a	Confirmed	
☐	☒	The exact sample size (*n*) for each experimental group/condition, given as a discrete number and unit of measurement
☐	☒	A statement on whether measurements were taken from distinct samples or whether the same sample was measured repeatedly
☐	☒	The statistical test(s) used AND whether they are one- or two-sided<br>*Only common tests should be described solely by name; describe more complex techniques in the Methods section.*
☐	☒	A description of all covariates tested
☐	☒	A description of any assumptions or corrections, such as tests of normality and adjustment for multiple comparisons
☐	☒	A full description of the statistical parameters including central tendency (e.g. means) or other basic estimates (e.g. regression coefficient) AND variation (e.g. standard deviation) or associated estimates of uncertainty (e.g. confidence intervals)
☐	☒	For null hypothesis testing, the test statistic (e.g. *F*, *t*, *r*) with confidence intervals, effect sizes, degrees of freedom and *P* value noted<br>*Give P values as exact values whenever suitable.*
☒	☐	For Bayesian analysis, information on the choice of priors and Markov chain Monte Carlo settings
☒	☐	For hierarchical and complex designs, identification of the appropriate level for tests and full reporting of outcomes
☒	☐	Estimates of effect sizes (e.g. Cohen's *d*, Pearson's *r*), indicating how they were calculated

Our web collection on statistics for biologists contains articles on many of the points above.

Software and code

Policy information about availability of computer code

Data collection	All clinical data were captured using the electronic data capture system RAVE.
Data analysis	SAS® Version 9.4 was used for all analyses.

For manuscripts utilizing custom algorithms or software that are central to the research but not yet described in published literature, software must be made available to editors and reviewers. We strongly encourage code deposition in a community repository (e.g. GitHub). See the Nature Portfolio guidelines for submitting code & software for further information.

Data

Policy information about availability of data

All manuscripts must include a data availability statement. This statement should provide the following information, where applicable:

- Accession codes, unique identifiers, or web links for publicly available datasets
- A description of any restrictions on data availability
- For clinical datasets or third party data, please ensure that the statement adheres to our policy

To ensure independent interpretation of clinical study results and enable authors to fulfill their role and obligations under the ICMJE criteria, Boehringer Ingelheim grants all external authors access to clinical study data pertinent to the development of the publication. In adherence with the Boehringer Ingelheim Policy on Transparency and Publication of Clinical Study Data, scientific and medical researchers can request access to clinical study data when it becomes available on

https://vivli.org/, and earliest after publication of the primary manuscript in a peer-reviewed journal, regulatory activities are complete, and other criteria are met. Please visit https://www.mystudywindow.com/msw/datasharing for further information.

Human research participants

Policy information about studies involving human research participants and Sex and Gender in Research.

Reporting on sex and gender	The findings apply to both sexes, sex was identified by self-report, and the analytical models included sex as a covariate, as described in the manuscript.
Population characteristics	Participants were men or women, 18 years of age or older, who had New York Heart Association functional class II–IV chronic heart failure and a left ventricular ejection fraction of more than 40%. The protocol required patients to have an N-terminal pro–B-type natriuretic peptide (NT-proBNP) level of more than 300 pg per milliliter or, for patients with atrial fibrillation at baseline, an NT-proBNP level of more than 900 pg per milliliter. Patients were excluded if they had a disorder that could change their clinical course, independent of heart failure, or if they had any condition that might jeopardize patient safety or limit their participation in the trial.
Recruitment	Patients were recruited as outpatients from the pool of heart failure patients from a given site, if fulfilling the inclusion/exclusion criteria. Any self-selection bias or other bias would be a function of the characteristics of patients presenting during the recruitment period and the judgement of the investigators, but would be very unlikely to be imbalanced between treatment arms, given the multicentre, double-blind, randomized trial design.
Ethics oversight	Ethics approval was obtained at each study site, and all patients provided informed consent to participate in the study

Note that full information on the approval of the study protocol must also be provided in the manuscript.

Field-specific reporting

Please select the one below that is the best fit for your research. If you are not sure, read the appropriate sections before making your selection.

☒ Life sciences ☐ Behavioural & social sciences ☐ Ecological, evolutionary & environmental sciences

For a reference copy of the document with all sections, see nature.com/documents/nr-reporting-summary-flat.pdf

Life sciences study design

All studies must disclose on these points even when the disclosure is negative.

Sample size	This was a secondary analysis of the EMPEROR-Preserved trial in which the following sample size calculation was performed, as previously reported (Anker SD, et al. N Engl J Med 2021;385:1451-1461). For this event-driven study, we determined that a target number of 841 adjudicated primary outcome events would provide 90% power to detect a hazard ratio of 0.8 for the primary outcome at a two-sided alpha level of 0.05. Assuming an annual 10% event rate in the placebo group, a recruitment period of 18 months, and a follow-up period of 20 months, we established a planned enrollment of 4126 patients, with the option of enrolling up to 6000 patients if the accumulation of primary outcome events was slower than expected. Accordingly, on the basis of monitoring of the primary outcome event rate during the trial, the number of patients who underwent randomization was increased to at least 5750, without any change in the target number of events. The increase in sample size was made without any knowledge of unblinded trial data.
Data exclusions	No data were excluded from the already published 5988 patients enrolled in EMPEROR-Preserved
Replication	Replication is not relevant as this is a single study in which key events (e.g. death) happened only once.
Randomization	In the original study, patient assignment to the treatment group was determined by a computer generated random sequence
Blinding	Patients, investigators and everyone involved in trial conduct or analysis or with any other interest in this double-blind trial remained blinded with regard to the randomised treatment assignments until after database lock.

Reporting for specific materials, systems and methods

We require information from authors about some types of materials, experimental systems and methods used in many studies. Here, indicate whether each material, system or method listed is relevant to your study. If you are not sure if a list item applies to your research, read the appropriate section before selecting a response.

Materials & experimental systems

n/a	Involved in the study
☒	☐ Antibodies
☒	☐ Eukaryotic cell lines
☒	☐ Palaeontology and archaeology
☒	☐ Animals and other organisms
☐	☒ Clinical data
☒	☐ Dual use research of concern

Methods

n/a	Involved in the study
☒	☐ ChIP-seq
☒	☐ Flow cytometry
☒	☐ MRI-based neuroimaging

Clinical data

Policy information about clinical studies

All manuscripts should comply with the ICMJE guidelines for publication of clinical research and a completed CONSORT checklist must be included with all submissions.

Clinical trial registration

NCT03057951

Study protocol

Study protocol included as supplementary materials

Data collection

Recruitment and follow-up was from March 27, 2017 until April 13, 2020 at 622 study sites in 23 countries, including hospitals, medical centres, clinics, and clinical research centres. Data was captured with an electronic case report form.

Outcomes

In this analysis the main outcomes were [i] the primary composite endpoint from the study of the time to a first event of hospitalization for heart failure (HHF) or cardiovascular death; [ii] first HHF; [iii] cardiovascular mortality; [iv] total (first and recurrent) HHF; and [v] the rate of change in the eGFR slope. The change in health-related quality of life was assessed using Kansas City Cardiomyopathy Questionnaire 23 (KCCQ-23).
These outcomes were pre-defined in the protocol, as follows.
5.1.1 Primary endpoint(s)
The composite primary endpoint for this trial is the time to first event of adjudicated CV death or adjudicated HHF in patients with Heart Failure with preserved Ejection Fraction (HFpEF).
5.1.2 Secondary endpoint(s)
The key secondary endpoints which are part of the testing strategy, are the following:
1. Occurrence of adjudicated HHF (first and recurrent),
2. eGFR (CKD-EPI)cr slope of change from baseline
Other secondary endpoints (not part of confirmatory testing hierarchy on trial level) are the following:
- Time to first occurrence of chronic dialysis or renal transplant or sustained* reduction of ≥40% eGFR (CKD-EPI)cr or
  - sustained eGFR (CKD-EPI)cr <15 mL/min/1.73 m2 for patients with baseline eGFR ≥30 mL/min/1.73 m2
  - sustained eGFR (CKD-EPI)cr <10 mL/min/1.73 m2 for patients with baseline eGFR <30 mL/min/1.73 m2
  *An eGFR (CDK-EPI)cr reduction is considered sustained, if it is determined by two or more consecutive post-baseline central laboratory measurements separated by at least 30 days (first to last of the consecutive eGFR values).
  Chronic dialysis is defined as dialysis with a frequency of twice per week or more often for at least 90 days.
- Time to first adjudicated HHF
- Time to adjudicated CV death
- Change from baseline in clinical summary score (HF symptoms and physical limitations domains) of the Kansas City Cardiomyopathy Questionnaire (KCCQ) at week 52
